# Claudin-1 Mediated Tight Junction Dysfunction as a Contributor to Atopic March

**DOI:** 10.3389/fimmu.2022.927465

**Published:** 2022-06-29

**Authors:** Yuhan Xia, Han Cao, Jie Zheng, Lihong Chen

**Affiliations:** Department of Dermatology, Ruijin Hospital, School of Medicine, Shanghai Jiaotong University, Shanghai, China

**Keywords:** atopic march, claudin-1, epithelial barrier, asthma, food allergy, atopic dermatitis

## Abstract

Atopic march refers to the phenomenon wherein the occurrence of asthma and food allergy tends to increase after atopic dermatitis. The mechanism underlying the progression of allergic inflammation from the skin to gastrointestinal (GI) tract and airways has still remained elusive. Impaired skin barrier was proposed as a risk factor for allergic sensitization. Claudin-1 protein forms tight junctions and is highly expressed in the epithelium of the skin, airways, and GI tract, thus, the downregulation of claudin-1 expression level caused by CLDN-1 gene polymorphism can mediate common dysregulation of epithelial barrier function in these organs, potentially leading to allergic sensitization at various sites. Importantly, in patients with atopic dermatitis, asthma, and food allergy, claudin-1 expression level was significantly downregulated in the skin, bronchial and intestinal epithelium, respectively. Knockdown of claudin-1 expression level in mouse models of atopic dermatitis and allergic asthma exacerbated allergic inflammation, proving that downregulation of claudin-1 expression level contributes to the pathogenesis of allergic diseases. Therefore, we hypothesized that the tight junction dysfunction mediated by downregulation of claudin-1 expression level contributes to atopic march. Further validation with clinical data from patients with atopic march or mouse models of atopic march is needed. If this hypothesis can be fully confirmed, impaired claudin-1 expression level may be a risk factor and likely a diagnostic marker for atopic march. Claudin-1 may serve as a valuable target to slowdown or block the progression of atopic march.

## Introduction

In the recent decades, the incidence of allergic diseases has continued to rise, affecting approximately 20% of the world’s population (1). Epidemiological studies have revealed an additive feature of atopic disorders, and the occurrence of food allergy (FA), allergic asthma (AA), and allergic rhinitis (AR) tends to increase after the onset of atopic dermatitis (AD) (2). The progression of allergic inflammation from the skin to the gastrointestinal (GI) tract, and then to airways, is termed “atopic march”. The mechanism of atopic march has still remained elusive, and skin barrier dysfunction was considered as a major contributor (3). It is noteworthy that other than the skin barrier, epithelial barriers in airways and GI tract also contribute to allergic sensitization process in atopic disorders (4). Tight junction proteins, including claidin-1, play a vital role in maintaining epithelial barriers of these organs (5). In the present study, we reviewed the involvement of epithelial barriers and tight junction proteins in allergic diseases, and hypothesized that claudin-1-mediated tight junction dysfunction could contribute to atopic march.

## The Atopic March

The atopic march refers to the developmental progression of allergic diseases from AD to FA, and then, to AA and AR. Proposed in 1923 by Cooke and Coca, the concept of “atopy” is closely associated with the elevated immunoglobulin E (IgE) production in various sites (6). Allergic conditions in the atopic march share a type 2 response phase, promoting the generation of allergen specific IgE. Other features of type 2 inflammation, such as edema, production of mucus, and activation of granulocytes were also observed in affected sites during the atopic march (7), indicating the progression of type 2 inflammation from the skin to the GI tract and airways.

As the first manifestation during atopic march, AD is a chronic inflammatory skin disease that occurs in infancy. More than 60% of children with AD developed eczema in the first 12 months of their life (8). Lesional skin of patients with AD is characterized by erythematous scaling plaques, eczema, xerosis and intense pruritus (9), with infiltration of type 2 innate lymphoid cells (ILC2s), T cells, eosinophils, and mast cells (10). The defected epithelial barrier and the predisposition to type 2 inflammation play an important role in the etiology of AD. For instance, the loss of functional mutations of FLG and SPINK5, which play important roles in skin barrier, were found to be closely associated with AD. Polymorphisms in the genes encoding interleukin-33 (IL-33) (11) and thymic stromal lymphopoietin (TSLP) (12), which trigger a type 2 response, predispose to AD. Environmental factors, such as toxins, irritants, and pollutants also contribute to the pathogenesis of AD (13). The incidence of atopic disorders tends to increase after the onset of AD. One recent study showed that 36.9% of children with severe AD developed asthma later on in their life (2), whereas the prevalence of AA in the general population was approximately 7.9% (14). Similarly, the incidence of FA was 40% in patients with AD (15), compared with a rate of 10% in the general population (16). Severe AD onset or a high serum level of IgE may predispose to atopic march development (17).

FA generally coexists with AD in children, and it is the most frequent cause of outpatient anaphylaxis (18). Other features of food allergy include abdominal pain, flatulence, vomiting, and diarrhea (19). Eosinophilic esophagitis (EoE) characterized by the chronic eosinophilic inflammation is closely correlated with food allergy (20). It was reported that sensitization mainly occurs before the food intake, suggesting the role of inflamed skin in sensitization (21). The exposure of skin to peanut dust raises the risk of developing peanut allergy (22). It is noteworthy that the occurrence of AA increases after the onset of FA (23). Patients with severe FA are at a higher risk of developing AA (24). Therefore, it is likely that the allergic inflammation in GI tract also contributes to the progression of atopic march.

AA and AR represent the endpoints of atopic march and generally occur at the age of 6–7 years old after the onset of AD (25). AA is characterized by chronic inflammation, remodeling and obstruction of the lower airways, while AR features chronic upper airway inflammation (26). Severe or early onset AD was reported to be associated with a stronger sensitization to inhalant allergens (27), which is central to the pathogenesis of AR and AA. The risk of AA is significantly elevated in patients with AR compared with the general population (28), suggesting the progression from AR to AA. Environmental factors, such as cigarettes and air pollutants were considered as determinants of this progression (29).

The mechanism of atopic march has still remained elusive. The defected skin barrier and increased alarmin production are the major contributors to the atopic march.

In particular, the loss of functional mutations of FLG, which encodes filaggrin (a protein that is indispensable for the integrity of stratum corneum), were found to be associated with five times increased progression from AD to FA (30). It was demonstrated that filaggrin dysfunction contributes to the atopic march through the increased allergen entry across an impaired skin barrier, which causes the skin sensitization to allergens (31). In response to the allergen entry, alarmins, such as TSLP, IL-25, and IL-33 are secreted by keratinocytes in skin (32). Alarmins trigger type 2 inflammation through interacting with basophils, mast cells, eosinophils, ILC2s, and Th2 cells. The secretion of alarmins can be amplified by the allergic inflammation, creating a positive feedback loop (33), thereby leading to the chronic inflammation of the skin. After entering the circulation, alarmins contribute to the systemic type 2 inflammation in the airways (34) and GI tract (35), and their roles in atopic march were previously verified in animal models of AD (36).

Importantly, although filaggrin is central to the integrity of skin barrier (37), it is absent on the bronchial or gut epithelial cells (38). Therefore, barrier components other than filaggrin shall be responsible for the progression of FA to AR and AA during the atopic march. In addition, it is essential to indicate whether the sensitization process in atopic march occurs in epithelium of the airways and the GI tract, as the epithelial barrier dysfunction in these organs contributes to the development of allergic diseases (39).

## The Epithelial Barrier in Atopic Disorders

The epithelial barrier generally consists of epithelial cells and interepithelial junctions. Interepithelial junctions are identified as tight junctions, intermediary junctions, and desmosomes that line between intercellular space following an apical order (40). Although epithelial barriers across organs vary in composition, they share the common role as the first line of defense against environmental exposures, such as allergens and pollutants. Epithelial barrier dysfunction caused by inherited or acquired factors contributes to the pathogenesis of AD, FA, AA, and AR.

### The Epidermal Barrier

The epidermal barrier in skin is composed of keratinocytes, junctional complexes, skin lipids, and microbiota. Keratinocytes express structural proteins, including filaggrin, loricrin, and hornerin, which maintain the intercellular cohesion of stratum corneum (41). Filaggrin deficiency mediated by FLG mutation or external irritants causes ILC2 expansion in skin, leading to AD onset (42). Skin lipids are synthesized and secreted by keratinocytes and sebaceous glands (43). Alteration of skin lipids, such as shortened molecular length of ceramides and free fatty acids, contributes to skin barrier defect in AD (44). Gut microbiome dysbiosis in AD is characterized by *Staphylococcus aureus* increase and *Staphylococcus epidermidis* impairment, contributing to the disease aggravation (45).

Tight junctions of the epidermal barrier include claudins, junctional adhesion molecules (JAMs), and zonula occludens (ZOs) (46). They regulate epithelial permeability and paracellular flux, preventing the entry of irritants and foreign antigens (47). Downregulation of claudin-1, claudin-4, and claudin-23 was reported in non-lesional skin of patients with AD (48), while single nucleotide polymorphisms in the CLDN-1 gene encoding claudin-1 were found to be associated with AD in several cohort studies (49, 50), suggesting the involvement of tight junctions in the pathogenesis of AD.

Adherens junctions in the epidermal barrier consist of cadherins and catenins, which cooperate with desmosomes to ensure a promising epidermal cohesion (51). Desmosomal adhesions in the epidermis are composed of desmogleins and corneodesmosin (52). Dysregulated desmosomal junctions contribute to AD development by interacting with skin microbiomes. Desmoglein-1 degradation by the cysteine protease of *Staphylococcus epidermidis* found on AD lesions contributes to exacerbation of skin damage (53). Aberrant display of corneodesmosin on the surface of corneocytes in AD supports colonization of *Staphylococcus aureus* (54), which increases disease severity. Moreover, the deficiency of corneodesmosin induces atopy development in peeling skin syndrome (55).

### The GI Tract Epithelial Barrier

The GI tract epithelial barrier comprises epithelial cells, intracellular junctions, the mucosal layer, and the intestinal microbiome (56). Cell components of this barrier vary across sites. The oral cavity, pharynx, and esophagus are lined with stratified squamous epithelium, containing non-keratinized keratinocytes, while the intestine is characterized by simple columnar epithelium lined with enteroclytes, goblet cells, Paneth cells, and M cells (57).

Secreted by goblet cells and enterocytes, the mucosal layer is a specific niche that contains the intestinal microbiota, protecting the epithelial barrier through its metabolic, immune, and trophic properties (58). The intestinal microbiota stimulates the Paneth cells, leading to the increased synthesis of antibacterial peptides and mucins, inhibiting the colonization of pathogenic bacteria (59). Additionally, the intestinal bacteria interact with the mucosal immune system and are indispensable for the maintenance of allergen tolerance by Treg cells (60). Short chain fatty acids secreted by gut microbiota confer protective effects against FA (61). In patients with FA, a less diverse intestinal microbiome with fewer *Bifidobacterium* and *Bacteroidetes* colonies could be observed before the onset of allergy (62). The deficiency of intestinal *Bifidobacterium* colonies was also found in patients with AD (63). Moreover, the intestinal microbiome with a low diversity during early infancy was reported to be associated with AA development at the age of 7 years old (64). It was demonstrated that the colonization of *Bifidobacterium* and *Lactobacillaceae* in the digestive tract and a more diverse intestinal microbiome prevent the development of allergic diseases (65), while intestinal colonization of *Bacteroidaceae* and *Clostridiaceae* predisposes to atopic disorders (66).

Tight junctions of the GI tract epithelium include occludin, claudins, ZO, JAMs, and zonulin. They are connected to the cytoskeleton and determine the permeability and selectivity of the epithelial layer (67). An increased intestinal permeability was found in patients with AD (68), AA (69), and FA (70). It was suggested that the dysregulation of claudin-1, claudin-4, claudin-5, and claudin-8 impairs intestinal barrier integrity and leads to the increased allergen penetration (71, 72). Moreover, the disruption of occludin raises intestinal permeability of macro-particles (73), which may contribute to the increased allergen entry. Low levels of esophageal zonulin-3, claudin-1, and claudin-7 have been detected in patients with EoE (74), a disease that is closely associated with food allergy. The hypoxia-inducible factor 1-alpha (HIF-1α)/claudin-1 axis was proposed as a therapeutic target for EoE (75) due to its critical role in maintaining barrier function.

Adherens junctions of the GI tract epithelium are made up of cadherins and catenins (76), while the desmosomal adhesions of the GI tract epithelium include desmogleins and desmocollins (77). Both the desmosomes and adherens junctions contribute to the regulation of paracellular permeability in the GI tract (78). In tissue specimens of patients with EoE, downregulation of desmoglein-1 was found, which could be reversed by the corticosteroid therapy (79).

### The Airway Epithelial Barrier

The airway epithelial barrier is mainly composed of ciliated cells, goblet cells, basal cells, junctional complexes, and the mucosal layer (80). Marked with a high number of cilia, ciliated cells constitute the major part of airway epithelium and are responsible for mucociliary clearance (81). Ciliary dysfunctions, such as shortened cilia and reduced ciliary beat frequency, are associated with disease severity in patients with AA (82) and AR (83). Goblet cells are secretory cells and produce mucus, a hydrogel gel that traps pathogens and inhaled particles (84). MUC5AC and MUC5B are the two major components of the mucus gel in normal airways (85). The balance between MUC5AC and MUC5B production is critical for mucociliary clearance and airway defense (86). In the AA pathogenesis, type 2 cytokines (e.g., IL-13 and IL-4) drive the excessive differentiation of goblet cells from basal cells *via* the Notch signaling pathway, leading to the dysregulated mucin secretion (87). A higher ratio of MUC5AC to MUC5B was found in the sputum of patients with AA and AR (88), and the increased proportion of MUC5AC is associated with impaired mucociliary clearance and airway plugging in AA development (89).

Tight junctions of the airway epithelium include occludin, claudins, ZOs, and JAMs, which regulate the permeablity of epithelial barrier. Reduced expression levels of claudin-18, claudin-4, claudin-1, and ZO-1 were found in the asthmatic bronchial epithelium (90, 91). External factors (e.g., respiratory syncytial virus) increase the risk of AA development by downregulating the expression levels of claudin-1 and occludin in the airways (92), while protease containing allergens directly cleave the tight junctions between cells, leading to barrier dysfunction (93, 94). Besides, the asthmatic inflammation leads to degradation of tight junctions. Co-culture with type 2 cytokines, such as IL-4 (95) and IL-13 (96), inhibits the expression levels of occludin, ZO-1, and claudin-1 in human primary bronchial epithelial cells, suggesting that the type 2 inflammation and the tight junction dysfunction lead to a vicious cycle in AA.

Adherens junctions of the airway epithelium include cadherins and catenins (97). Reduced expression levels of a-catenin and e-cadherin were found in the airway epithelial cells from asthmatic patients (91). Protease containing allergens from pollens or house dust mite could downregulate the expression level of e-cadherin in human airway epithelial cells (98). Moreover, co-culture with IL-4 and IL-13 inhibits the expression levels of a-catenin and e-cadherin in human primary bronchial epithelial cells (96).

Taken together, the epithelial barrier dysfunction in skin, GI tract, and airways contributes to the development of atopic disorders through increased allergen entry and allergic sensitization. On the other hand, the allergic inflammation damages the epithelial barriers, leading to a vicious circle in allergic diseases. Such interaction may occur across different sites, as the intestinal dysbiosis contributes to AD development, while the systemic type 2 response in AD damages the intestinal barrier function. Furthermore, epithelial tight junction dysfunction contributes to the development of atopic disorders. Particularly, we found that the expression level of claudin-1 in epithelial cells is commonly downregulated in allergic diseases of the skin, lungs, and GI tract.

## The Role of Claudin-1 in Atopic Disorders

**Figure 1:**
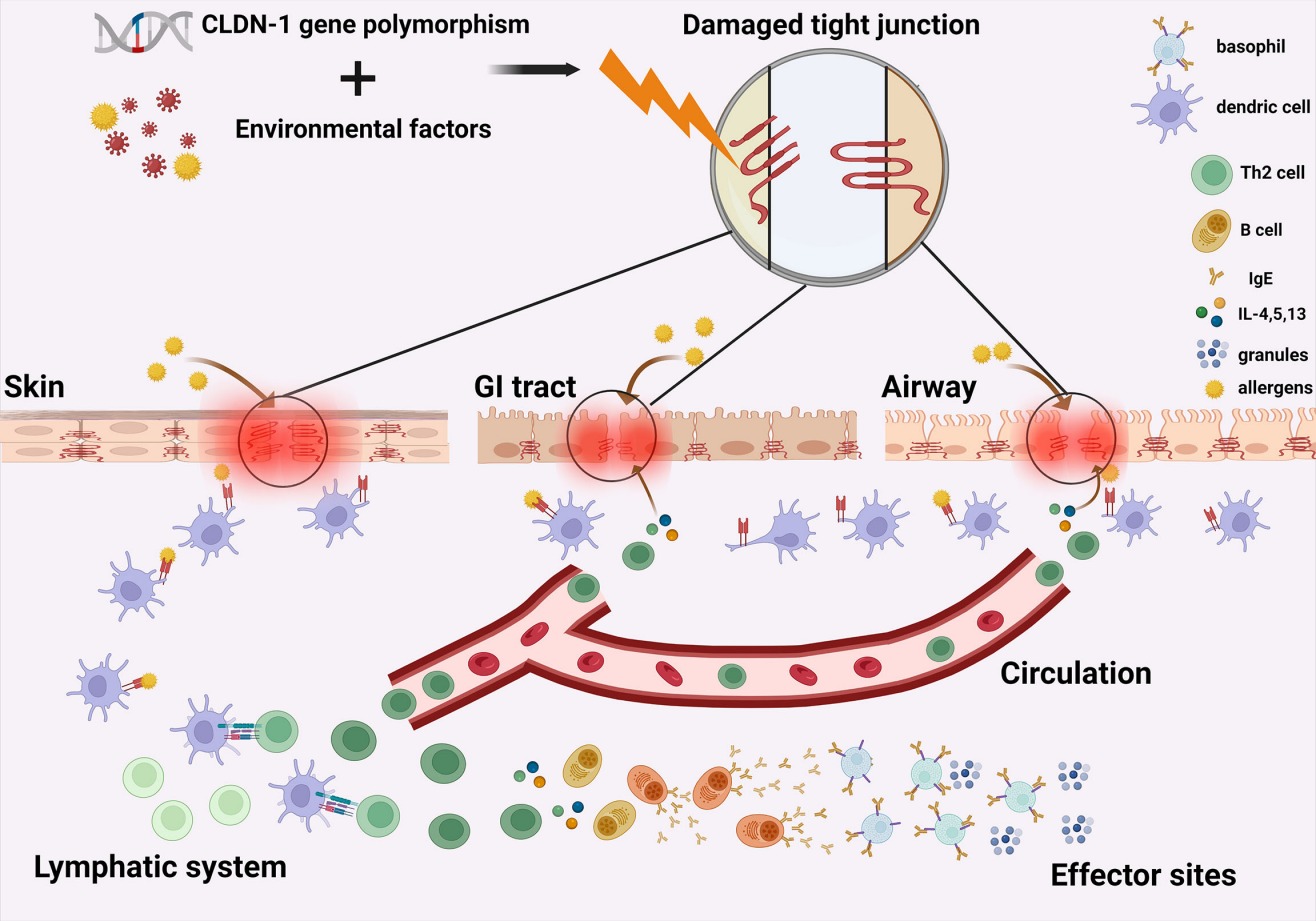
Claudin-1-mediated tight junction dysfunction contributes to the atopic march. The synergic effect of CLDN-1 gene polymorphism and environmental factors lead to common disturbance of claudin-1 in the epithelium of skin, airways and GI tract, causing tight junction dysfunction and impaired epithelial barrier function in these organs. Entry of allergens through damaged skin barrier leads to the systemic type 2 inflammation in patients with AD, which further downregulates the claudin-1 expression level in the GI tract and airways. This exacerbation of barrier impairment in the intestinal and airway epithelium finally leads to the progression of allergic inflammation from skin to the GI tract and then to airways. GI, gastrointestinal; Th2 cell, Type 2 helper T cell; B cell, B lymphocyte; IgE, immunoglobulin E; IL-4, interleukin 4; IL-5, interleukin-5; IL-13, interleukin-13. Granules, granules secreted by basophils containing mediators of inflammation.

Claudin-1, a member of the claudin family, plays an important role in barrier function, not only in skin (99), but also in airways (100) and GI tract (101) (**Figure 1**). Coded by CLDN1, claudin-1 forms the tight junctions between the stratum granulosum and stratum corneum (102). It can also be detected at high levels in esophagus, liver, and gall bladder (103).

Mutation of CLDN1 causes a systemic disorder (104). Missense mutation of CLDN1 is associated with neonatal ichthyosis-sclerosing cholangitis syndrome, which has been confirmed by several clinical observations (105). Patients with this autosomal dominant inherited disorder could be accompanied with ichthyosis, leucocytic vacuoles, and sclerosing cholangitis (106), suggesting the central role of claudin-1 in both skin and GI tract barrier function.

The reduced expression level of claudin-1 may cause tight junction dysfunction (107), and it has been suggested to be a key mechanism for allergic diseases of the skin (108), airways (109), and GI tract (110). In patients with AD, impaired skin barrier function mediated by downregulated claudin-1 has been reported (111). In patients with AA, bronchial claudin-1 expression level is negatively correlated with disease severity (109). Moreover, in patients with FA, a decreased expression level of claudin-1 has been found in the oral mucosa (110), esophagus (112), and intestines (113), and has been associated with the increased production of IgE (113), a key component for allergic inflammation.

Moreover, the expression level of claudin-1 in the GI tract (114, 115) and airways (95, 116) can be downregulated by IL-4, IL-5, and IL-13 that present in patients with AD and FA, suggesting the involvement of claudin-1 in the progression of atopic inflammation to other sites.

Regarding the downregulation of claudin-1 expression level in epithelial cells of patients with allergic diseases of the skin, airways and GI tract, as well as the important role of claudin-1 in epithelial barrier function, we attempted to clarify the potential role of claudin-1 in the progression of atopic march.

## Statement of Hypothesis

Claudin-1-mediated tight junction dysfunction contributes to the atopic march (**Figure 1**).

Specifically, the synergic effect of CLDN-1 gene polymorphism and environmental factors, including lifestyles (117), infections (118) or pollutants (119) mediate the downregulation of claudin-1 in the epithelium of skin, airways, and GI tract, causing epithelial barrier dysfunction in these organs. Entry of allergens through damaged skin barrier leads to the systemic type 2 inflammation in patients with AD, which further downregulates the claudin-1 expression level in the GI tract and airways. This exacerbation of barrier impairment in the intestinal and airway epithelium causes increased allergen sensitization at these sites and finally leads to the progression of allergic inflammation from skin to the GI tract and then to airways.

## Evaluation of the Hypothesis

The hypothesis, “claudin-1-mediated tight junction dysfunction contributes to atopic march”, is mainly supported by three lines of evidence: clinical observations, animal models of allergic diseases, and CLDN-1 knockdown studies.

Clinical observations and animal models suggested that claudin-1 was downregulated in the skin, airway, and GI epithelium in patients with AD, AA, and FA.

In the skin lesions of patients with AD, the expression level of caudin-1 was drastically reduced. CLDN1 expression was negatively correlated with severity of skin inflammation, while positively correlated with the skin barrier function and a dose-dependent relationship was observed (120). Polymorphism of CLDN-1 was associated with susceptibility to AD in case control studies (121), and was responsible for the increased production of IgE after exposure of skin to mold (122), contributing to the allergic sensitization (123).

Moreover, in mouse models of AD, claudin-1 expression level in skin tissue was significantly downregulated, and was correlated with hallmarks of dermal inflammation, such as the increased epidermal thickness, altered keratinocyte differentiation, increased keratinocyte proliferation, and impaired barrier function (124).

In the GI tract, small intestinal biopsy specimens of patients with food allergy showed significant downregulation of claudin-1 (125), whereas patients with food allergy to profilin presented with drastically reduced claudin-1 expression level in oral mucosa, which allowed profilin to penetrate into the oral epithelial barrier, leading to allergic sensitization (110). The claudin-1 expression level was significantly downregulated in the esophagus of patients with EoE, an allergic disease associated with food antigens (74). A food allergen challenge induced a rapid degradation of intestinal intercellular junction proteins, including claudin-1, in a mouse model of food allergy (126). Downregulation of CLDN1 expression level in the GI tract was also associated with an increased serum IgE level (127).

Aside from the skin and GI tract, in the airway epithelium, claudin-1 expression level was negatively correlated with asthma severity, both in patients with asthma and in the house dust mite-induced mouse asthmatic model. Additionally, claudin-1 expression level was significantly downregulated in bronchial epithelial cells in asthmatic children compared with that in healthy controls in response to viral stimulation (92, 128). Neutrophil autophagy and neutrophil extracellular traps could enhance asthma severity by inducing claudin-1 degradation (129).

**Figure 2:**
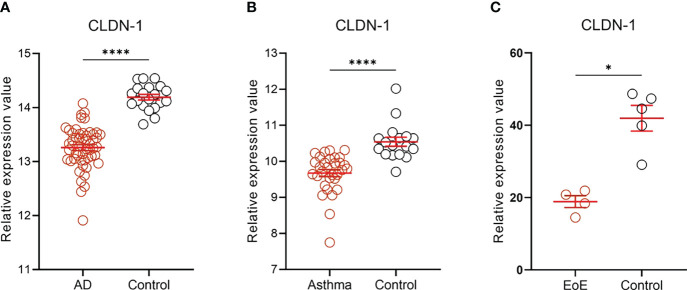
CLDN-1 expression level in the epithelial tissue of patients with allergic diseases. CLDN-1 expression level was downregulated in the epithelium of skin, airways, and GI tract in patients with AD, asthma, and EoE, respectively. **(A)** CLDN-1 mRNA expression in lesional skin of AD patients (n=52) and healthy controls (n = 20). **(B)** CLDN-1 mRNA expression in bronchial epithelium of patients with severe asthma (n = 31) and healthy controls (n = 17). **(C)** CLDN-1 mRNA expression in esophageal epithelium of patients with EoE (n = 4) and healthy controls (n = 5). * P < 0.05; **** P < 0.0001 by Mann-Whitney U test. Error bars indicate SEM. AD, atopic dermatitis; EoE, eosinophilic esophagitis.

We analyzed the publicly available RNA-seq datasets of epithelial tissue samples from patients with AD (GSE130588), asthma (GSE43696), and food allergy (GSE113341). Strikingly, the commonly significant downregulation of CLDN-1 expression level was found in the epidermis of patients with AD (**Figure 2A**), in bronchial epithelium of patients with AA (**Figure 2B**), and in esophageal epithelium of patients with EoE (**Figure 2C**), suggesting that claudin-1 expression level is commonly downregulated in epithelial cells of patients with allergic diseases of the skin, airways, and GI tract.

Studies on CLDN1 knockdown were conducted using mouse models of AD and AA. CLDN1 knockdown in mice caused epithelial barrier dysfunction and morphological features of AD in the skin, including hyperkeratosis, acanthosis, and neutrophil infiltration were mediated by an innate immune response (130). While the increased expression level of claudin-1 in skin tissue alleviated atopic symptoms in an AD mouse model, providing a potential therapeutic approach for AD (131).

CLDN1 knockdown in murine lungs significantly exacerbated the airway inflammation and the increased airway hyperreactivity in a mouse model of asthma (109), linking the claudin-1 downregulation directly to AA pathogenesis through the dysregulated airway barrier function. However, the restoration of claudin-1 expression level in the airways significantly suppressed bronchial hyperresponsiveness and decreased serum IgE level in an asthmatic mouse model (132).

Upregulation of claudin-1 expression level strengthened the epithelial barrier, lowering the risk of FA development (133).

The above-mentioned studies suggested a common correlation between claudin-1 expression level and allergic diseases of the skin, airways, and GI tract. Besides, a direct pathogenetic role of claudin-1 knockdown in AD and AA was confirmed, suggesting that downregulation of claudin-1 expression level contributes to the pathogenesis of these allergic diseases.

However, the evidence only demonstrated separate correlations between claudin-1 expression level and allergic diseases of the skin, airways, and GI tract.

To demonstrate that downregulation of claudin-1 expression level contributes to the progression of allergic inflammation from the skin to the airways and GI tract in patients with AD, clinical data concerning the claudin-1 expression level in the airways and GI tract of patients with atopic march should be acquired. If a lower claudin-1 expression level could be found in the airway and GI tract of AD patients with atopic march than in cases without atopic march, we can be accordingly more certain that claudin-1 expression level contributes to the progression of atopic march.

Organ-specific, that is, airway- and GI tract-specific claudin-1 knockdown in mouse models of atopic march, might be an excellent approach to prove that downregulation of claudin-1 expression level in the airways and gut epithelial barrier accelerates atopic march, thereby contributing to atopic march.

## Discussion

The concept of atopic march was proposed based on the clinical observations that patients with AD were more susceptible to AA and FA (134). The true nature of this connection, however, is still controversial, with alarmins commonly secreted by epithelial cells in various organs as likely candidates (135).

Skin barrier dysfunction mediated by filaggrin mutation was found to be associated with the increased allergen passage through skin and a systemic allergic response, suggesting that dysfunction of the skin epithelial barrier is crucial for allergic sensitization (31). However, filaggrin is not expressed in the airways and GI tract, restricting filaggrin-mediated sensitization to the skin.

Importantly, the tight junctions formed by the claudins are also essential for the integrity of the epithelial barrier (136). In contrast to filaggrin, claudins are highly expressed in the epithelium of airways and GI tract, thus, the downregulation of claudins could mediate a common dysregulation of the epithelial barrier function in these organs, potentially leading to allergic sensitization at various sites.

In addition, in patients with AD, AA, and FA, claudin-1 expression level is significantly downregulated in the epidermis, bronchial epithelium, and GI tract, respectively.

Knockdown of claudin-1 expression level in the mouse models of AD (130) and AA (109) significantly exacerbated allergic inflammation, while the upregulation of claudin-1 expression level restored epithelial barrier function and decreased the severity of allergic diseases in these mouse models (131, 133), proving that downregulation of claudin-1 expression level contributes to the pathogenesis of allergic inflammation.

Furthermore, the epithelial claudin-1 expression level in GI tract (114) and airways (128, 137) can be downregulated by the systemic type 2 inflammation in patients with AD and FA. This downregulation of claudin-1 expression level causes epithelial barrier dysfunction, increased allergen entry and allergen sensitization in GI tract and airways, and may lead to the development of FA and AA, suggesting the role of claudin-1 expression level in the progression of atopic march.

Therefore, our hypothesis that downregulation of claudin-1 expression level contributes to the atopic march is well supported with separate evidence concerning each of the involved allergic diseases. To further validate the hypothesis, evidence of downregulation of claudin-1 expression level in the airways and GI tract from patients with atopic march is needed. Airway- and GI tract-specific knockdown of CLDN1 in a mouse model of atopic march can also be a great approach to prove our hypothesis.

If the hypothesis that the tight junction dysfunction caused by downregulation of claudin-1 expression level contributes to the atopic march can be fully confirmed, impaired CLDN1 expression level may be a risk factor and likely a diagnostic marker for atopic march. Claudin-1 may be a valuable target to slowdown or even block the progression of atopic march.

## Data Availability Statement

The datasets presented in this study can be found in online repositories. The names of the repository/repositories and accession number(s) can be found in the article/supplementary material.

## Author Contributions

YX and HC have written and conceptualized the article. JZ and LC has contributed to the writing and revision process. All authors contributed to the article and approved the submitted version.

## Funding

This work was supported by grants from the National Natural Science Foundation of China (81803134).

## Conflict of Interest

The authors declare that the research was conducted in the absence of any commercial or financial relationships that could be construed as a potential conflict of interest.

## Publisher’s Note

All claims expressed in this article are solely those of the authors and do not necessarily represent those of their affiliated organizations, or those of the publisher, the editors and the reviewers. Any product that may be evaluated in this article, or claim that may be made by its manufacturer, is not guaranteed or endorsed by the publisher.
